# Establishment of epigenetic patterns in development

**DOI:** 10.1007/s00412-012-0365-x

**Published:** 2012-03-17

**Authors:** Martin Leeb, Anton Wutz

**Affiliations:** Wellcome Trust Centre for Stem Cell Research, University of Cambridge, Tennis Court Road, Cambridge, CB2 1QN UK

## Abstract

The distinct cell types of the body are established from the fertilized egg in development and assembled into functional tissues. Functional characteristics and gene expression patterns are then faithfully maintained in somatic cell lineages over a lifetime. On the molecular level, transcription factors initiate lineage-specific gene expression programmmes and epigenetic regulation contributes to stabilization of expression patterns. Epigenetic mechanisms are essential for maintaining stable cell identities and their disruption can lead to disease or cellular transformation. Here, we discuss the role of epigenetic regulation in the early mouse embryo, which presents a relatively well-understood system. A number of studies have contributed to the understanding of the function of Polycomb group complexes and the DNA methylation system. The role of many other chromatin regulators in development remains largely unexplored. Albeit the current picture remains incomplete, the view emerges that multiple epigenetic mechanisms cooperate for repressing critical developmental regulators. Some chromatin modifications appear to act in parallel and others might repress the same gene at a different stage of cell differentiation. Studies in pluripotent mouse embryonic stem cells show that epigenetic mechanisms function to repress lineage specific gene expression and prevent extraembryonic differentiation. Insights into this epigenetic “memory” of the first lineage decisions help to provide a better understanding of the function of epigenetic regulation in adult stem cell differentiation.

## Introduction

All cells of an organism are generated from a single fertilized oocyte during development. Cell differentiation is guided by transcription factors that define expression profiles of intermediate precursors and the functional differentiated cell types of the organs and tissues. Mutual antagonism between lineage-specific transcription factors is thought to establish distinct cell-type-specific expression patterns (for a detailed discussion see Graf and Enver 2009). This view is supported by evidence showing that expression of certain transcription factors can enforce a dominant cell fate. Demonstrated cell fate conversions include the generation of muscle cells from fibroblasts through expression of the transcription factor Myf5 or the conversion of B cells into macrophages by C/EBPα expression (Graf and Enver 2009). These findings are consistent with an instructive role of transcription factors in the establishment of cell fates.

In addition to transcription factor networks, other mechanisms act to stabilize cell fates once these have been established. These mechanisms include small RNAs and chromatin- or DNA-modifying protein complexes. In mammals, DNA cytosine methylation and chromatin-modifying complexes of the Polycomb group (PcG) proteins are prominent examples for regulators that repress genes that are inappropriate for a certain cell type or lineage. Thereby the developmental potential of cells within a lineage becomes progressively restricted in differentiation. The stabilizing effect of epigenetic restrictions becomes apparent when differentiated cells are reprogrammed to pluripotent stem cells through the expression of a cocktail of transcription factors (Orkin and Hochedlinger 2011). Generating induced pluripotent stem (iPS) cells is an inefficient and lengthy process that possibly highlights the opposing action of epigenetic regulation against cell-type reprogramming (Yamanaka 2009).

When and how epigenetic patterns are established in development is a focus of current research. Here, we discuss the present understanding of epigenetic regulation in the early mouse embryo where a large number of studies have characterized the establishment of different cell lineages.

## The lineages of the early embryo

**Fig. 1:**
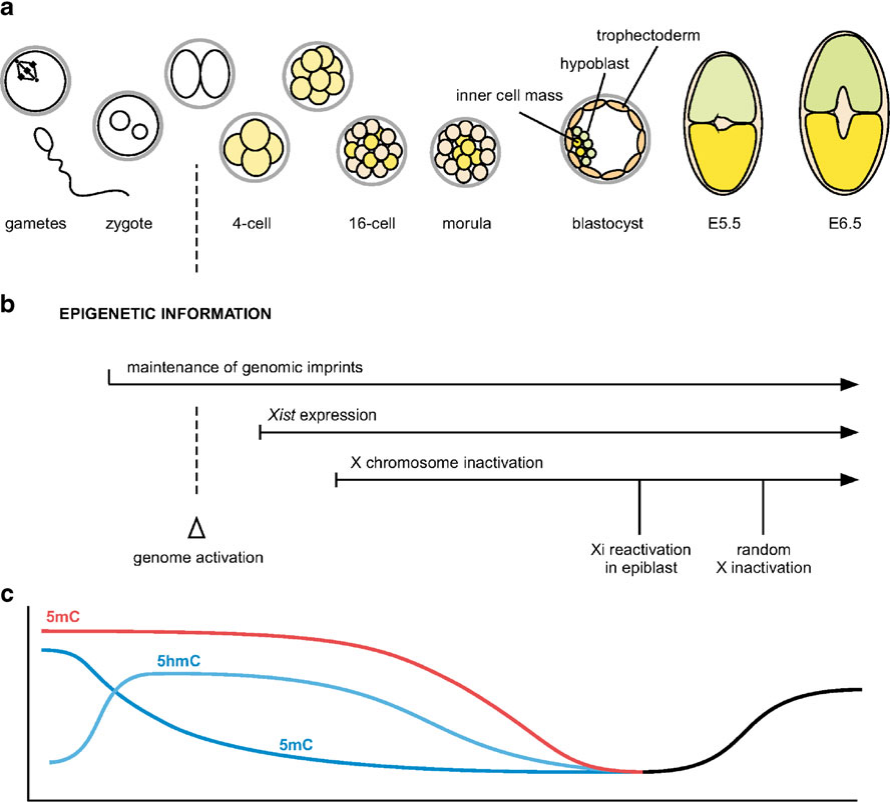
Epigenetic regulation in early mouse development. **a** Schematic representation of mouse development is aligned with key epigenetic events in panels b and c. The trophectoderm (TE) lineage is the first to differentiate from cells that have an outside position of morula stage embryos (red shading). At the blastocyst stage, the hypoblast (*green*) is specified. Inner cell mass cells (*yellow*) will give rise to the developing mouse embryo whereas TE and hypoblast form extraembryonic tissues. **b** Genomic imprints are parent-of-origin-specific marks that are maintained during embryogenesis and regulate the differential expression of the maternal and paternal copy of imprinted genes. X chromosome inactivation and reactivation is observed during development of female embryos. **c** A diagram illustrating global changes in DNA methylation (*5mC*) and DNA hydroxymethylation (*5hmC*) levels. In cleavage stage embryos the paternal (*blue lines*) and maternal (*red*) genomes are differentially marked by *5hmC* and *5mC*, respectively. Both 5mC and 5hmC levels decrease during development to the blastocyst stage and then 5mC increases as the embryonic lineages are formed

A number of studies have contributed to the understanding of patterning and lineage commitment in the mouse embryo (Arnold and Robertson 2009). Expression of the zygotic genome commences at the two-cell stage (Fig. 1a, Hamatani et al. 2004). The trophectoderm (TE) is the first lineage to differentiate forming the outside cells of the morula stage embryo (for review see Sasaki 2010). Position-dependent Hippo signalling activates the transcriptional activity of Tead4 in cells with an outer position in the embryo. Tead4 induces *Cdx2* and *Gata3* expression and thereby initiates TE specification (Ralston et al. 2010). Differentiation of the TE is then regulated by a transcription factor network including Eomes and Elf5. In contrast, the inner cell mass (ICM) cells that give rise to the embryo express Oct4, Sox2 and Nanog. Antagonism between the transcription factor networks around Cdx2 and Oct4, which each positively regulate their own transcription, reinforces the separation of two distinct fates (Niwa et al. 2005). The ICM develops into the epiblast and hypoblast (primitive endoderm; PE) which are determined by transcription factors Nanog and Gata6, respectively and are further regulated by MAPK (mitogen-activated protein kinase) signalling (Chazaud et al. 2006). It has been shown that the formation of the hypoblast can be suppressed by culturing mouse embryos with inhibitors of the MAPK pathway (Nichols et al. 2009), whereas *Nanog* is required to establish a pluripotent cell fate (Silva et al. 2009). Thus, repeated bifurcations of cell fates caused by position-dependent cell signalling and transcriptional feedback loops initiate the formation of the lineages of the early embryo.

In addition to transcription factors, epigenetic mechanisms contribute to the regulation of gene expression in the early embryo (Fig. 1b). A number of imprinted genes are expressed from a single parental allele. Genomic imprinting implies that information for expression or repression of a parental allele must be maintained from the maternal and paternal germlines throughout fertilization and development. Since diffusible transcription factors have equal access to both alleles, genomic imprinting is best explained by a mechanism that links gene regulatory information to the DNA or chromatin of the gene locus (Ferguson-Smith 2011). Epigenetic regulation in *cis* is also highlighted by the process of X inactivation in female embryos (Augui et al. 2011). Thereby, the X-linked gene dosage is equalized to one active X chromosome between male (XY) and female (XX) cells. In mice, inactivation of the paternally inherited X chromosome is initiated at the four-cell stage (Fig. 1b). Imprinted inactivation of the paternal X chromosome is maintained in the extraembryonic lineages, whereas reactivation of the inactive X chromosome (Xi) is observed in the cells of the ICM that give rise to the epiblast. In the embryonic lineages, dosage compensation is re-established at the time of gastrulation by random inactivation of either the maternally or paternally inherited X chromosome. Notably, neither X inactivation nor genomic imprinting are essential for development to the blastocyst stage. Uniparental diploid mouse embryos such as parthenogenotes and androgenotes have been observed to reach the blastocyst stage and even to implant. Similarly, disruption of X inactivation by deletion of the *Xist* gene arrests development only after implantation (Marahrens et al. 1997). Furthermore, combined disruption of imprinting and X inactivation in haploid parthenogenotes can be compatible with blastocyst development and recently it has been shown that haploid embryonic stem cells can be established and maintained in culture (Elling et al. 2011; Leeb and Wutz 2011).

The function of epigenetic regulation has also been elucidated in different stem cell lines of the early embryo that reflect characteristics of different lineages (Rossant 2007, Fig. 2a). Trophoblast stem (TS) cells and extraembryonic endoderm stem (XEN) cells maintain the capacity to differentiate into the trophoblast and primitive endoderm (PE) lineage, respectively. In both stem cell types, imprinted inactivation of the paternal X chromosome is observed (Kunath et al. 2005). Mouse embryonic stem (ES) cells are derived from the ICM of the blastocyst. ES cells maintain the potential for differentiating into all cell types of the embryo, which has been impressively demonstrated by using tetraploid aggregation (Nagy et al. 1990) or injection of ES cells into eight-cell embryos (Poueymirou et al. 2007) to generate mice whose cells are almost entirely derived from ES cells. In contrast to their wide lineage potential, ES cells do normally not contribute to extraembryonic tissues (TE and PE) consistent with characteristics of cells from the ICM of the late blastocyst and early epiblast. A second pluripotent stem cell type can be obtained from the mouse postimplantation epiblast (Brons et al. 2007; Tesar et al. 2007). Similar to ES cells, these epiblast-derived stem cells (EpiSCs) possess a wide lineage differentiation potential in culture. However, EpiSCs do not efficiently contribute to embryogenesis when injected into blastocysts. Furthermore, EpiSCs have initiated X inactivation whereas female mouse ES cells possess two active X chromosomes (Guo et al. 2009). This indicates that the two pluripotent cell types are distinguished by developmental and epigenetic characteristics. It is interesting to observe that pluripotent stem cells established from most mammals including humans resemble the EpiSC type with the notable exception of the rat (Blair et al. 2011). This could potentially indicate that the EpiSC cell state is maintained more stably in evolution. The developmentally restricted differentiation potential of the stem cells of the early mouse embryo has been studied for understanding transcriptional and epigenetic regulation in lineage specification.

**Fig. 2:**
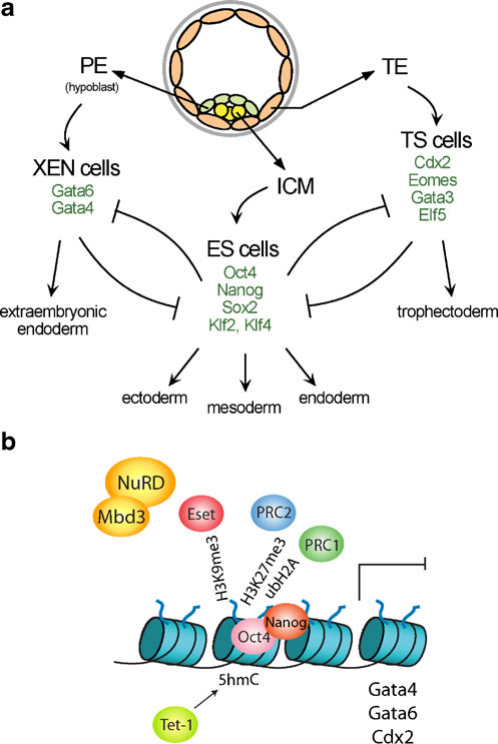
Transcriptional control and epigenetic regulation in the lineages of the mouse blastocyst. **a** The three lineages of the blastocyst can give rise to stem cell lines in culture. Transcription factor networks as observed in trophectoderm stem (*TS*) cells, extraembryonic endoderm stem (*XEN*) cells and *ES cells* are shown (*green*) and their mutual antagonistic regulation is indicated. **b** Repression of key transcription factors of extraembryonic lineage development in ES cells has been analysed. A number of epigenetic regulators contribute to repress genes and their activity and interactions with chromatin on gene promoters are summarized in the scheme

## The function of epigenetic regulators during early embryogenesis

In mammals, a number of chromatin- and DNA-modifying activities have been identified and their function in development and differentiation has been characterized (Table 1). DNA methylation and chromatin-modifying activities of the Polycomb group complexes have been extensively studied and are presently best understood. A role of other epigenetic regulators such as the histone methylases G9a and Eset as well as the DNA hydroxymethylase Tet family of proteins have also been implicated in development (Fig. 2b). Albeit the current knowledge of epigenetic pathways in mammals is not yet complete the available data provides insights into the distinct roles of epigenetic regulation in development.

**Table 1:** Epigenetic modifications and their regulation and function in mouse development

Modification	Enzyme/factor	Genomic target	Function	Phenotype of mutation
5mC	Dnmt1	CpG island promoters genomic repeats	Maintenance DNA methyltransferase	Embryonic lethal at midgestation and disruption of imprints
	Dnmt3a	Promoters	De novo methylation	Postnatal lethality
	Dnmt3b	Pericentric repeats promoters	De novo methylation	Embryonic lethality after E9.5
	Dnmt3L	Imprinted genes	Recruitment of Dnmts in germline and early embryo	Imprinting disruption and failure of gametogenesis
	Uhrf1/Np95	Hemimethylated DNA	Recruits Dnmt1 to hemimethylated DNA	Embryonic lethal after gastrulation
	SmcHD1	Inactive X chromosome	Maintenance of gene repression and DNA methylation on Xi	Female embryonic lethality at midgestation
	Combined mutation of Dnmt1, Dnmt3a and Dnmt3b causes loss of genomic 5mC and is compatible with ES cell survival and extraembryonic development, but not with survival of differentiated embryonic cell types.			
5hmC	Tet1	Promoters	Hydroxymethylation	No phenotype
	Tet2	Promoters	Hematopoietic differentiation	Tet2 mutation causes enhanced hematopoietic progenitor survival and leukaemia
	Tet3	Paternal genome	5hmC modification of paternal genome in preimplantation embryo	Loss of early postimplantation stage embryos
	TDG	5hmC modified DNA	Demethylation by base excision repair pathway	Embryonic lethality before E12.5
	Oxidation of 5hmC by the Tet1-4 enzymes is thought to enable demethylation of DNA through base excision by thymidine deglycosylase (TDG) and subsequent repair.			
H3K27me3	PRC2 (Ezh2, Suz12 and Eed)	Gene promoters LTR transposons	Repression of developmental and cell cycle regulators	Lethality after implantation
H2AK119ub	PRC1 (Ring1b and Ring1a)	Gene promoters	Repression of developmental and cell cycle regulators	Ring1b mutation causes gastrulation arrest
	Polycomb complexes (PRC1 and PRC2) maintain gene repression of developmental control genes including Hox gene clusters. They also act on other targets such as the cell cycle regulator p16. Depending on the gene mutation of either complex or combined, loss of PRC1 and PRC2 functions lead to derepression.			
H3K9me3	ESet	Gene promoters	Gene repression and viral repression	Early embryonic lethality
	G9a/GLP	Gene promoters retrotransposons	Gene and transposon repression and DNA methylation	Embryonic lethality
	Suv39h1 and Suv39h2	Pericentric heterochromatin	Maintenance of heterochromatin and genomic stability	Viable but genomic instability due to compromised centromere function

### Polycomb complexes

PcG proteins are transcriptional repressors that have a role in maintaining repression of developmental regulator genes from plants to mammals (Beisel and Paro 2011). Their evolutionary origin can be traced back to unicellular organisms (Shaver et al. 2010). In animals, PcG proteins are widely known for their function in *Hox* gene regulation. PcG proteins are components of chromatin-modifying complexes. Polycomb repressive complex 1 (PRC1) catalyses monoubiquitinylation of histone H2A (ubH2A) and PRC2 mediates di- and tri-methylation of histone H3 lysine 27 (H3K27me2 and H3K27me3). Gene deletion studies in mice have shown that both PRC1 and PRC2 are essential for development and their disruption leads to arrest soon after implantation (O'Carroll et al. 2001; Voncken et al. 2003; Wang et al. 2001). Consistent with normal development to the blastocyst stage, ES cells have been obtained that are deficient in PRC1 or PRC2 catalytic activity (Chamberlain et al. 2008; Leeb and Wutz 2007; Schoeftner et al. 2006). Surprisingly, these ES cells have the ability to differentiate into embryonic lineages despite aberrant gene expression is observed including misregulation of the *Hox* gene clusters (Chamberlain et al. 2008; Leeb and Wutz 2007). A combined disruption of PRC1 and PRC2 is not compatible with differentiation suggesting an overlapping function of both PcG complexes in cell differentiation (Leeb et al. 2010). Consistent with this idea PRC1 and PRC2 cooperate to repress a number of target genes including *Cdx2*, *Gata4*, *Gata6* and *Sox7* (Leeb et al. 2010).

PcG regulation in mammals is complex and the number of PcG genes has expanded during evolution. Especially, the PRC1 component Ring1b participates in multiple complexes that show a heterogeneous and cell-type-specific composition. As a consequence, deletions of individual PcG genes result in complex phenotypes. Whereas deletion of *Eed* and *Ring1b* in ES cells appears to eliminate the catalytic functions of PRC2 and PRC1, respectively, deletions in *Ring1a*, *Bmi1* or *Mel18* have less severe consequences (Schuettengruber et al. 2007). A partial compensation for the loss of *Ring1b* function by *Ring1a* has also been described (de Napoles et al. 2004; Stock et al. 2007). Other proteins have been identified that contribute to the recruitment of PcG complexes or modulate their function. Jarid2 has been identified as a stoichiometric component of the PRC2 complex in ES cells (Herz and Shilatifard 2010). Jarid2 has DNA binding activity and modulates PRC2 recruitment to target genes (Li et al. 2010). However, mutation of *Jarid2* does not lead to an upregulation of PcG target genes (Landeira et al. 2010). Loss of *Jarid2* can been associated with either a loss (Li et al. 2010; Pasini et al. 2010) or increase (Peng et al. 2009; Shen et al. 2009) of H3K27me3 on different target genes. Complex regulation is also suggested by the finding that the enzymatic activity of PRC1 is not essential for chromatin compaction and gene repression within the *Hoxb* locus (Eskeland et al. 2010). The PcG system involves heterogeneous complexes that distinctly regulate a wide range of different target genes and whose function is essential after specification of the early embryonic lineages.

### DNA methylation

In mammals, methylated cytosine is predominantly observed in the context of CpG dinucleotides and is involved in a range of processes including embryogenesis, genomic imprinting and tumorigenesis (Bird 2002). CpG methylation is generally correlated with transcriptional inactivity of promoters. In mice, three DNA methyltransferases Dnmt1, Dnmt3a and Dnmt3b have been identified (Li et al. 1992; Okano et al. 1999). Gene deletion studies have provided insight into the function of DNA methylation in development (Table 1). Disruption of *Dnmt1* results in lethality before E 10.5 (Li et al. 1992). Similarly, loss of *Dnmt3b* is lethal before E 9.5 (Okano et al. 1999). Although deletion of *Dnmt3a* is compatible with embryonic development, *Dnmt3a*-deficient mice die within the first weeks after birth (Okano et al. 1999). Interestingly, the specification and function of early embryonic lineages is largely unaffected by loss of DNA methyltransferases. Deficiency for all three DNA methyltransferases in ES cells leads to a complete loss of DNA methylation (Tsumura et al. 2006). These ES cells show increased cell death in differentiation and do not contribute to embryonic lineages at E 10.5 when injected into blastocysts. However, they can colonize the ICM and show limited ability to contribute to chimeric E 8.5 embryos. Notably, the extraembryonic lineages can be established in the absence of DNA methylation, whereas maintenance of differentiated embryonic cell types is dependent on the DNA methylation system (Sakaue et al. 2010).

Maintenance of DNA methylation patterns is mediated by the restoration of symmetrical methylation on hemimethylated CpG dinucleotides following DNA replication. This is facilitated by the Np95/Uhrf1 protein that recruits Dnmt1 to hemimethylated CpG sites (Bostick et al. 2007; Sharif et al. 2007). *Np95* is required for maintaining DNA methylation patterns and its disruption in mice leads to lethality after gastrulation (Sharif et al. 2007). Thus, deletion of *Np95* and *Dnmt1* appear to cause similar phenotypes.

Methylated cytosine in DNA can be further modified by the Tet family of proteins that catalyse the oxidation of 5-methylcytosine (5mC) to 5-hydroxymethylcytosine (5hmC) (Tahiliani et al. 2009). Tet1 is highly expressed and specific for ES cells (Wu et al. 2011). Tet1 binds preferentially to CpG-rich sequences at promoters and its activity has been associated with both activating and repressing functions. It has been suggested that 5hmC could potentially mask the silencing effect of 5mC. In addition, 5hmC appears to be involved in the repression of Polycomb-targeted developmental regulators (Wu et al. 2011). In ES cells, Tet1-repressed genes include *Sox17*, *Gata6* and *Cdx2*. In line with these observations, disruption of *Tet1* expression predisposes to differentiation into extraembryonic tissues (Ficz et al. 2011; Ito et al. 2010). A recent study of *Tet1*-deficient mice has shown that *Tet1* is dispensable for maintaining pluripotency and its loss is compatible with embryonic and postnatal development (Dawlaty et al. 2011). This could possibly point towards compensation by other Tet family proteins. Recently, it has been shown that *Tet3* is required for hydroxymethylation of the paternal genome in zygotes (Gu et al. 2011). In mouse cleavage stage embryos, the paternal genome is largely devoid of 5mC but enriched in 5hmC, whereas the maternal genome is marked by 5mC (Iqbal et al. 2011, Fig. 1c). Loss of maternal *Tet3* leads to increased developmental failure after implantation. Both 5hmC and 5mC can be further oxidized by the Tet dioxygenases to 5-carboxylcytosine (5acC), which in turn is removed by thymine-DNA glycosylase thereby establishing a mechanism for DNA demethylation (He et al. 2011). These findings suggest a complex chemistry leading to different modification states of cytosine in DNA and allowing methyl-removal to unmethylated DNA (Nabel and Kohli 2011). Similar to chromatin modifications, DNA methylation has to be understood as a dynamic and reversible modification in development.

### Chromatin-modifying complexes

A large number of chromatin-modifying proteins have been identified in mice. These contribute to constitutive heterochromatin on centromeres and telomeres and also have roles in silencing genomic repeat elements. The formation of heterochromatin at the pericentric regions as well as the variation and propagation of epigenetic states to a dynamic chromatin template have been extensively studied (reviewed in Fodor et al. 2010). In addition, several histone methylases and deacetylases function in development. The histone methyltransferases G9a and Eset catalyse histone H3 lysine 9 methylation and contribute to gene regulation in the early embryo. Deletion of *G9a* results in embryonic lethality between E 8.5 and E 9.5 (Tachibana et al. 2002). Mutation of *Eset* results in peri-implantation lethality between E 3.5 and E 5.5. Consistent with this early lethality, *Eset*-deficient ES cells could not be established (Dodge et al. 2004) indicating an essential function for *Eset* in preimplantation development. Eset mediates di- and tri-methylation of histone H3 lysine 9 (H3K9me2/3) (Lohmann et al. 2010). However, genetic ablation of *Eset* function does not result in a global reduction of H3K9me3, which is possibly explained by the fact that the majority of H3K9me3 is associated with pericentric heterochromatin which depends on the Suv39h1/2 histone methyltransferases (Fodor et al. 2010). Deletion of *Eset* results in a failure to establish the epiblast due to inappropriate trophectodermal differentiation (Yeap et al. 2009). The demonstration that *Eset*-depleted ES cells can incorporate into the trophectoderm and differentiate into placental tissues shows that the lineage restriction of ES cells towards extraembryonic cell fates depends on Eset function. One of the Eset target genes in ES cells is *Cdx2* (Lohmann et al. 2010) and an interaction between Eset with Oct4 has been reported (Yeap et al. 2009; Yuan et al. 2009). These findings suggest that Oct4 might recruit Eset for repression of genes involved in extraembryonic differentiation. Consistent with this interpretation, loss of Oct4 and Eset in ES cells are associated with overlapping phenotypes. A critical role of Eset in epiblast development also explains the inability to establish *Eset*-deficient ES cells (Yeap et al. 2009).

The NuRD complex has also been implicated in the establishment of the epiblast lineage. NuRD is a multi subunit complex that possesses nucleosome remodelling and histone deacetylase (HDAC) activity. Interference with NuRD function by deletion of the *Mbd3* gene abrogates the formation of a normal epiblast possibly caused by inappropriate trophectodermal differentiation (Kaji et al. 2007). However, *Mbd3*-deficient ES cells have been derived suggesting that NuRD contributes to but is not absolutely essential for epiblast establishment. *Mbd3*-deficient ES cells can self renew in the absence of LIF signalling (Kaji et al. 2006). It has been proposed that in ES cells Mbd3 has an additional function and might facilitate the exit of pluripotency and entry into differentiation. Taken together, these findings suggest that Eset and Mbd3 might directly influence the formation of the epiblast lineage by preventing extraembryonic differentiation. This could hint at the molecular basis of a “memory” of earlier lineage decisions. Alternatively, these epigenetic regulators might act as co-repressors together with transcription factors such as the interaction of Eset with Oct4 suggests. Recently, a genome-wide study has shown that Tet1 (5hmC) and Mbd3 colocalize in ES cells (Yildirim et al. 2011). Mbd3 and Tet1 recruitment to many target loci is interdependent indicating an additional function of Mbd3 in regulating patterns of DNA hydroxymethylation.

### Apparent robustness of early embryos towards epigenetic disruptions

Studies of loss of function situations in mice have contributed to the understanding of epigenetic regulators in early embryonic lineage decisions. In some cases, the formation of blastocysts and implantation were not perturbed by the loss of epigenetic regulators. However, phenotypic consequences of loss of epigenetic regulators could be masked by stable transcription factor networks that would keep cell fates largely intact. This idea is supported by the observation in cultured ES cells that are deficient in Polycomb complex activity. These cells can be maintained in culture even if a large set of genes are misregulated which is advantageous for investigating epigenetic regulators as cell viability is not affected. However, several aspects should be considered when interpreting data in the early embryo. In the preimplantation embryo, a delayed phenotype can potentially also be caused due to partial compensation by a maternal store of proteins and RNA in the oocytes (Hamatani et al. 2004). An example is Ezh2 which is detected as a maternally inherited protein in oocytes (Erhardt et al. 2003). Loss of Ezh2 in oocytes causes growth retardation of neonates even when zygotic expression from the paternal allele restores Ezh2 protein at the 4-cell stage. This illustrates a distinct function for Ezh2 in oocytes and preimplantation embryos. Compensation for phenotypic effects could also result from the parallel action of more than one epigenetic system. In this case, the phenotype would only be observed if simultaneous mutations would be examined. At present, redundancies between different chromatin-modifying activities are not completely understood but some could be predicted from overlapping chromatin targets. In addition, a recent report suggests that many epigenetic marks remain unknown and are still to be explored (Tan et al. 2011). Notably, despite of potential compensation, substantial disruptions in gene regulation are observed in embryos with mutations in epigenetic regulators (Leeb et al. 2010). Taken together with the observation that a lack of dosage compensation or loss of imprinting can be tolerated by the early mouse embryo, it is tempting to speculate that the early cell lineages are characterized by a robustness for maintaining crucial features of cell types and morphology. At later stages of development, a multitude of cell interactions are required for maintaining viability and morphology. This makes it more difficult to clearly discern the effects of epigenetic disruptions on gene regulation within tissues and organs. The early embryonic lineages might, thus, provide a suitable model to unravel the molecular interactions between transcription and chromatin regulation for obtaining a framework for the interpretation of experiments in more complex systems.

## Establishment of epigenetic patterns

For exploring the targets of epigenetic regulation, genome-wide analyses of binding sites and chromatin modifications have been performed in ES cells. More than 2,000 gene promoters have been found to be bound by PcG proteins (Boyer et al. 2006; Ku et al. 2008; Mikkelsen et al. 2007). PcG targets include a number of lineage-specific transcription factors suggesting that PcG complexes maintain the multilineage differentiation potential of ES cells by preventing activation of differentiation genes. In ES cells, nearly all PcG target genes are in a bivalent chromatin configuration comprising tri-methylation of histone H3 lysine 27 and lysine 4. Furthermore, RNA polymerase II is found in a non-processive state (Boyer et al. 2006; Ku et al. 2008; Mikkelsen et al. 2007). It has been suggested that this special chromatin configuration allows rapid activation of repressed PcG target genes by developmental cues when cells enter differentiation into a lineage (Stock et al. 2007). Indeed upon differentiation, the bivalent state is resolved to either an H3K27me3- or a H3K4me3-only state (Bracken et al. 2006; Mohn et al. 2008). Activation of PcG-repressed target genes involves the Trithorax (TrxG) group of proteins (Schuettengruber et al. 2007). Recently, a number of additional activities have been reported that act in the removal of PcG complexes from chromatin. The displacement of PcG proteins and activation of target genes has been reported to be dependent on serine 28 phosphorylation of histone H3 (Gehani et al. 2010). In addition, demethylation of H3K27me3 is catalysed by the UTX demethylase (Seenundun et al. 2010), and the deubiquitination of ubH2A is catalysed by PR-DUB (Scheuermann et al. 2010). These findings indicate that the PcG/TrxG targets are regulated by multiple positive and negative regulators that facilitate the establishment and removal of PcG repression. PcG patterns are established in a dynamic and lineage-specific manner in development (Bracken et al. 2006; Mikkelsen et al. 2007; Mohn et al. 2008), suggesting that keeping a fine balance between different chromatin regulators is key to faithfully maintain specific expression patterns.

Genome-wide analysis of DNA methylation in ES cells (Meissner et al. 2008) has shown little overlap with PcG target genes suggesting that DNA methylation patterns are specified independently of PcG complexes. A gain of DNA methylation was observed during differentiation, consistent with an early developmental phenotype of mutations in DNA methyltransferases (Mohn et al. 2008, Fig. 1c). Promoters of several hundred genes become cytosine methylated in lineage-committed progenitor cells. However, only few differences between neural progenitors and terminally differentiated neurons were detected. This observation suggests that methylation of non-lineage genes is already established at the progenitor state where it might act to restrict the differentiation potential to within the lineage. Interestingly, many genes that are PcG targets in ES cells acquire DNA methylation during differentiation indicating a function of DNA methylation in maintaining the inactive promoter state when genes are not activated in a certain lineage (Mohn et al. 2008). It is conceivable that PcG proteins act in early stages to establish reversible repression and DNA methyltransferases stabilize gene repression at a later stage of differentiation. A similar mechanisms has been proposed in X inactivation where initiation of chromosome-wide silencing is independent of DNA methylation. However, maintenance of gene repression on the Xi in somatic cells requires DNA methylation (Blewitt et al. 2008; Sado et al. 2000). DNA methylation is also required for regulation of imprinted genes (Li et al. 1993). In addition, *Elf5* is an important target of DNA methylation in ES cells. Demethylation of *Elf5* has been shown to induce trophectodermal differentiation (Hemberger et al. 2009; Ng et al. 2008). However, DNA methylation is neither essential for the self renewal of ES cells nor for nuclear reprogramming of somatic cells to a pluripotent state (Pawlak and Jaenisch 2011).

In contrast to DNA methylation which is underrepresented on PcG target promoters, 5hmC shows a pronounced overlap with bivalent PcG-bound chromatin (Williams et al. 2011). A functional overlap between the 5hmC system and PcG-mediated gene repression has also been inferred from data showing that more than 40 % of genes derepressed after *Tet1* depletion are also upregulated in *Eed*-deficient cells. However, co-binding to the same targets appears not to be due to a direct biochemical interaction between Tet and PcG proteins (Pastor et al. 2011). Notably, *Tet1* also has an activating role and overlaps with H3K36me3 on active genes (Wu et al. 2011) suggesting an independent role of 5hmC in priming genes for activation by maintaining reversible repression in ES cells by protecting CpG dinucleotides from aberrant DNA methylation (Wu et al. 2011). In support of this idea, it has been demonstrated that depletion of *Tet1* leads to methylation of the *Nanog* promoter in ES cells. Taken together, these findings suggest multiple roles for Tet proteins in modulating gene repression and DNA methylation.

**Fig. 3:**
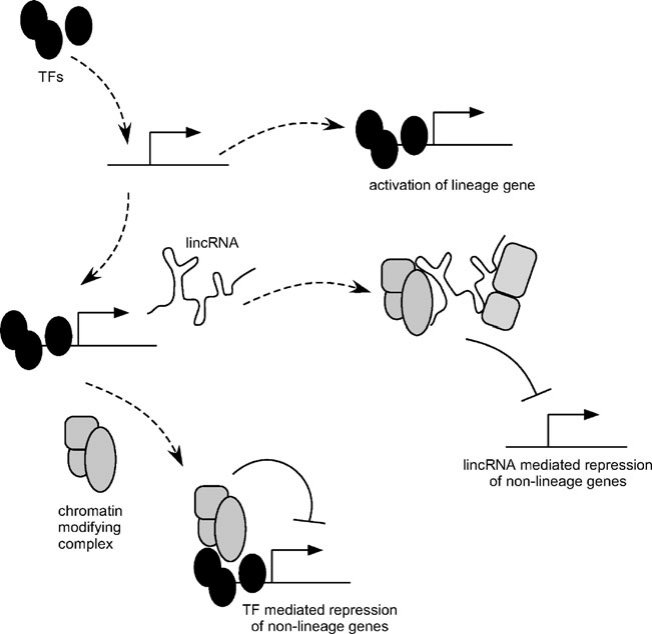
Transcription factors, chromatin-modifying complexes and lincRNAs have been implicated in specifying epigenetic patterns. Transcription factors (*TFs*) activate genes in a cell-type- or lineage-specific manner. In addition TFs also associate with chromatin- or DNA-modifying activities that repress certain of their target genes. Among TF-activated genes are a class of long noncoding RNAs (*LincRNAs*) that can bind different chromatin regulators and might function to target them to certain genomic regions. Thereby, lincRNAs provide a mechanism for establishing epigenetic patterns on regions that do not have binding sites for TFs. Repressive chromatin marks are important for preventing activation of genes associated with other lineages thereby preventing aberrant differentiation

Albeit transcription factor binding sites are associated with promoters of several PcG target genes the establishment of epigenetic patterns can hardly be explained by this overlap alone. It appears that multiple mechanisms specify chromatin and DNA modifications (Fig. 3). DNA methylation patterns appear independent of transcription factor networks in ES cells and might reflect maintenance of silencing of genes that have become repressed long ago, such as imprinted genes that have derived their marking from one of the parental germlines. However, this general pattern does not rule out that sequence elements specify the methylation state. Indeed, it has been shown that DNA sequence elements can specify hypo- or de novo methylation in a developmental context (Lienert et al. 2011). For some genes, it is conceivable to construct a logic series from transcription factors to PcG complexes and on to DNA methylation. However, binding of PcG proteins is only partially explained by the binding of core transcription factors in ES cells. This suggests that other mechanisms specify epigenetic patterns—the underlying DNA sequence and 3D chromosome compartmentalization have been suggested. In addition, noncoding RNAs have been observed to contribute to PcG recruitment. Prominent examples are HOTAIR in the regulation of the human *HOXD* gene cluster (Rinn et al. 2007) and *Xist* in X inactivation (Mak et al. 2002; Plath et al. 2003). A recent study has systematically investigated the function of a large number of noncoding RNAs in ES cells (Guttman et al. 2011). Several of these lincRNAs bind chromatin-modifying proteins and repress lineage programmes. Notably, the majority of lincRNAs are regulated by ES cell transcription factors which suggest that RNAs could constitute an important class of regulators for establishing epigenetic patterns in ES cells.

## Heritability of epigenetic marks

Establishment of an epigenetic memory requires a mechanism to perpetuate marks during cell division in such a way that they can be inherited by the descendants within a cell lineage. How defined patterns of chromatin modifications are copied to both replicated DNA strands is presently not fully understood. A mechanism for maintaining DNA methylation patterns has been suggested. Uhrf1/NP95 binds to hemimethylated CpG sites and recruits Dnmt1 thereby re-establishing cytosine methylation on the replicated DNA strand. It is easy to see how symmetrically methylated CpG sites can be maintained by this mechanism. For histone modifications, the situation is more complex. Firstly, the large variety of histone modifications and their combinations suggest that these marks need to be established by multiple complexes in a sequential manner. Secondly, histones become displaced during replication from the DNA strands. Therefore, reassembly of modified histones into nucleosomes would require precise control mechanisms. Thirdly, there is evidence suggesting that histone modifications are not intrinsically stable. An example is the constant requirement of *Xist* RNA for recruitment of PcG complexes to the Xi (Kohlmaier et al. 2004; Pullirsch et al. 2010; Schoeftner et al. 2006). This could point to a scenario whereby patterns of modified histones are specified by complex mechanisms. This leads to the question: What constitutes a memory mark?

Some work suggests that DNA methylation patterns and maintenance of hypoacetylated chromatin might be a stable and heritable configuration (Blewitt et al. 2008; Csankovszki et al. 2001; Pullirsch et al. 2010). Hypoacetylation of chromatin could be maintained by recruitment of HDACs which have been reported to interact with Dnmt1 (Fuks et al. 2000). A function of HDACs in silencing of PcG target genes has also been established (Schuettengruber et al. 2007), suggesting that multiple interactions between chromatin-modifying activities could provide stability and contribute to the maintenance of repressed chromatin. This view is supported by observations that different histone modifications frequently overlap on important developmental genes and detailed models for the maintenance of pericentric heterochromatin have been developed (Fig. 2b, Fodor et al. 2010). Mutual reinforcement of silencing by multiple mechanisms could provide increased stability and also facilitate modulation by cell signalling in development. Future research into the structure of chromatin will certainly lead to a better understanding of this important aspect of gene regulation.

## Conclusion and future outlook

The early embryo provides insights into the function of epigenetic mechanisms in cell differentiation. Epigenetic modifications appear to have a role in restricting the differentiation potential of cells. How and to which extent chromatin modulators interact to maintain gene expression states remains largely unexplored. It is becoming increasingly clear that master regulators of lineage identity are co-regulated by several epigenetic silencing mechanisms in ES cells. Regulators of extraembryonic lineages including Gata4, Gata6 and Cdx2 are derepressed in PRC1-, PRC2-, *Mbd3*-, *Tet1*- and *Eset*-deficient ES cells. Thus, disruption of single epigenetic systems leads to upregulation of extraembryonic core transcription factors, and, in some cases, aberrant differentiation into extraembryonic tissues. The extent to which chromatin regulators and the DNA methylation machinery have overlapping functions and how these interact in development will be an important question for future research.

Notably, ES cell self-renewal can occur in the absence of certain epigenetic regulators despite substantial misregulation of gene expression. Whilst it appears that epigenetic regulators perform a function in noise reduction in the gene expression profile of ES cells, their function becomes critical upon differentiation for restricting expression programmmes. This consideration is also important for understanding epigenetic regulation in adult stem cell systems. Recent studies have begun to address the function of PcG proteins in adult stem cells. Notably, in the hematopoietic system, disruption of PRC1 and PRC2 was found associated with distinct and opposing outcomes in stem cell proliferation and differentiation (Majewski et al. 2010). This unexpected result highlights the complexity of blood lineage formation where homeostatic feedback regulation of proliferation and differentiation contributes to the observed phenotypes. In the future, it will be important to construct a theoretical framework of epigenetic regulation for better understanding cell-type changes during tissue repair and reprogramming.

## References

[CR1] Arnold SJ, Robertson EJ (2009). Making a commitment: cell lineage allocation and axis patterning in the early mouse embryo. Nat Rev Mol Cell Biol.

[CR2] Augui S, Nora EP, Heard E (2011). Regulation of X-chromosome inactivation by the X-inactivation centre. Nat Rev.

[CR3] Beisel C, Paro R (2011). Silencing chromatin: comparing modes and mechanisms. Nat Rev.

[CR4] Bird A (2002). DNA methylation patterns and epigenetic memory. Genes Dev.

[CR5] Blair K, Wray J, Smith A (2011). The liberation of embryonic stem cells. PLoS Genet.

[CR6] Blewitt ME, Gendrel AV, Pang Z, Sparrow DB, Whitelaw N, Craig JM, Apedaile A, Hilton DJ, Dunwoodie SL, Brockdorff N, Kay GF, Whitelaw E (2008). SmcHD1, containing a structural-maintenance-of-chromosomes hinge domain, has a critical role in X inactivation. Nat Genet.

[CR7] Bostick M, Kim JK, Esteve PO, Clark A, Pradhan S, Jacobsen SE (2007). UHRF1 plays a role in maintaining DNA methylation in mammalian cells. Science (New York, NY).

[CR8] Boyer LA, Plath K, Zeitlinger J, Brambrink T, Medeiros LA, Lee TI, Levine SS, Wernig M, Tajonar A, Ray MK, Bell GW, Otte AP, Vidal M, Gifford DK, Young RA, Jaenisch R (2006). Polycomb complexes repress developmental regulators in murine embryonic stem cells. Nature.

[CR9] Bracken AP, Dietrich N, Pasini D, Hansen KH, Helin K (2006). Genome-wide mapping of Polycomb target genes unravels their roles in cell fate transitions. Genes Dev.

[CR10] Brons IG, Smithers LE, Trotter MW, Rugg-Gunn P, Sun B, de Sousa C, Lopes SM, Howlett SK, Clarkson A, Ahrlund-Richter L, Pedersen RA, Vallier L (2007). Derivation of pluripotent epiblast stem cells from mammalian embryos. Nature.

[CR11] Chamberlain SJ, Yee D, Magnuson T (2008). Polycomb repressive complex 2 is dispensable for maintenance of embryonic stem cell pluripotency. Stem cells (Dayton, Ohio).

[CR12] Chazaud C, Yamanaka Y, Pawson T, Rossant J (2006). Early lineage segregation between epiblast and primitive endoderm in mouse blastocysts through the Grb2-MAPK pathway. Dev Cell.

[CR13] Csankovszki G, Nagy A, Jaenisch R (2001). Synergism of Xist RNA, DNA methylation, and histone hypoacetylation in maintaining X chromosome inactivation. J Cell Biol.

[CR14] Dawlaty MM, Ganz K, Powell BE, Hu YC, Markoulaki S, Cheng AW, Gao Q, Kim J, Choi SW, Page DC, Jaenisch R (2011). Tet1 is dispensable for maintaining pluripotency and its loss is compatible with embryonic and postnatal development. Cell Stem Cell.

[CR15] de Napoles M, Mermoud JE, Wakao R, Tang YA, Endoh M, Appanah R, Nesterova TB, Silva J, Otte AP, Vidal M, Koseki H, Brockdorff N (2004). Polycomb group proteins Ring1A/B link ubiquitylation of histone H2A to heritable gene silencing and X inactivation. Dev Cell.

[CR16] Dodge JE, Kang YK, Beppu H, Lei H, Li E (2004). Histone H3-K9 methyltransferase ESET is essential for early development. Mol Cell Biol.

[CR17] Elling U, Taubenschmid J, Wirnsberger G, O'Malley R, Demers SP, Vanhaelen Q, Shukalyuk AI, Schmauss G, Schramek D, Schnuetgen F, von Melchner H, Ecker JR, Stanford WL, Zuber J, Stark A, Penninger JM (2011). Forward and reverse genetics through derivation of haploid mouse embryonic stem cells. Cell Stem Cell.

[CR18] Erhardt S, Su IH, Schneider R, Barton S, Bannister AJ, Perez-Burgos L, Jenuwein T, Kouzarides T, Tarakhovsky A, Surani MA (2003). Consequences of the depletion of zygotic and embryonic enhancer of zeste 2 during preimplantation mouse development. Development.

[CR19] Eskeland R, Leeb M, Grimes GR, Kress C, Boyle S, Sproul D, Gilbert N, Fan Y, Skoultchi AI, Wutz A, Bickmore WA (2010). Ring1B compacts chromatin structure and represses gene expression independent of histone ubiquitination. Mol Cell.

[CR20] Ferguson-Smith AC (2011). Genomic imprinting: the emergence of an epigenetic paradigm. Nat Rev.

[CR21] Ficz G, Branco MR, Seisenberger S, Santos F, Krueger F, Hore TA, Marques CJ, Andrews S, Reik W (2011). Dynamic regulation of 5-hydroxymethylcytosine in mouse ES cells and during differentiation. Nature.

[CR22] Fodor BD, Shukeir N, Reuter G, Jenuwein T (2010). Mammalian Su(var) genes in chromatin control. Annu Rev Cell Dev Biol.

[CR23] Fuks F, Burgers WA, Brehm A, Hughes-Davies L, Kouzarides T (2000). DNA methyltransferase Dnmt1 associates with histone deacetylase activity. Nat Genet.

[CR24] Gehani SS, Agrawal-Singh S, Dietrich N, Christophersen NS, Helin K, Hansen K (2010). Polycomb group protein displacement and gene activation through MSK-dependent H3K27me3S28 phosphorylation. Mol Cell.

[CR25] Graf T, Enver T (2009). Forcing cells to change lineages. Nature.

[CR26] Gu TP, Guo F, Yang H, Wu HP, Xu GF, Liu W, Xie ZG, Shi L, He X, Jin SG, Iqbal K, Shi YG, Deng Z, Szabo PE, Pfeifer GP, Li J, Xu GL (2011). The role of Tet3 DNA dioxygenase in epigenetic reprogramming by oocytes. Nature.

[CR27] Guo G, Yang J, Nichols J, Hall JS, Eyres I, Mansfield W, Smith A (2009). Klf4 reverts developmentally programmed restriction of ground state pluripotency. Development.

[CR28] Guttman M, Donaghey J, Carey BW, Garber M, Grenier JK, Munson G, Young G, Lucas AB, Ach R, Bruhn L, Yang X, Amit I, Meissner A, Regev A, Rinn JL, Root DE, Lander ES (2011). lincRNAs act in the circuitry controlling pluripotency and differentiation. Nature.

[CR29] Hamatani T, Carter MG, Sharov AA, Ko MS (2004). Dynamics of global gene expression changes during mouse preimplantation development. Dev Cell.

[CR30] He YF, Li BZ, Li Z, Liu P, Wang Y, Tang Q, Ding J, Jia Y, Chen Z, Li L, Sun Y, Li X, Dai Q, Song CX, Zhang K, He C, Xu GL (2011). Tet-mediated formation of 5-carboxylcytosine and its excision by TDG in mammalian DNA. Science (New York, NY).

[CR31] Hemberger M, Dean W, Reik W (2009). Epigenetic dynamics of stem cells and cell lineage commitment: digging Waddington's canal. Nat Rev Mol Cell Biol.

[CR32] Herz HM, Shilatifard A (2010). The JARID2-PRC2 duality. Genes Dev.

[CR33] Iqbal K, Jin SG, Pfeifer GP, Szabo PE (2011). Reprogramming of the paternal genome upon fertilization involves genome-wide oxidation of 5-methylcytosine. Proc Natl Acad Sci USA.

[CR34] Ito S, D'Alessio AC, Taranova OV, Hong K, Sowers LC, Zhang Y (2010). Role of Tet proteins in 5mC to 5hmC conversion, ES-cell self-renewal and inner cell mass specification. Nature.

[CR35] Kaji K, Caballero IM, MacLeod R, Nichols J, Wilson VA, Hendrich B (2006). The NuRD component Mbd3 is required for pluripotency of embryonic stem cells. Nat Cell Biol.

[CR36] Kaji K, Nichols J, Hendrich B (2007). Mbd3, a component of the NuRD co-repressor complex, is required for development of pluripotent cells. Development.

[CR37] Kohlmaier A, Savarese F, Lachner M, Martens J, Jenuwein T, Wutz A (2004). A chromosomal memory triggered by Xist regulates histone methylation in X inactivation. PLoS Biol.

[CR38] Ku M, Koche RP, Rheinbay E, Mendenhall EM, Endoh M, Mikkelsen TS, Presser A, Nusbaum C, Xie X, Chi AS, Adli M, Kasif S, Ptaszek LM, Cowan CA, Lander ES, Koseki H, Bernstein BE (2008). Genomewide analysis of PRC1 and PRC2 occupancy identifies two classes of bivalent domains. PLoS Genet.

[CR39] Kunath T, Arnaud D, Uy GD, Okamoto I, Chureau C, Yamanaka Y, Heard E, Gardner RL, Avner P, Rossant J (2005). Imprinted X-inactivation in extra-embryonic endoderm cell lines from mouse blastocysts. Development.

[CR40] Landeira D, Sauer S, Poot R, Dvorkina M, Mazzarella L, Jorgensen HF, Pereira CF, Leleu M, Piccolo FM, Spivakov M, Brookes E, Pombo A, Fisher C, Skarnes WC, Snoek T, Bezstarosti K, Demmers J, Klose RJ, Casanova M, Tavares L, Brockdorff N, Merkenschlager M, Fisher AG (2010). Jarid2 is a PRC2 component in embryonic stem cells required for multi-lineage differentiation and recruitment of PRC1 and RNA Polymerase II to developmental regulators. Nat Cell Biol.

[CR41] Leeb M, Wutz A (2007). Ring1B is crucial for the regulation of developmental control genes and PRC1 proteins but not X inactivation in embryonic cells. J Cell Biol.

[CR42] Leeb M, Wutz A (2011). Derivation of haploid embryonic stem cells from mouse embryos. Nature.

[CR43] Leeb M, Pasini D, Novatchkova M, Jaritz M, Helin K, Wutz A (2010). Polycomb complexes act redundantly to repress genomic repeats and genes. Genes Dev.

[CR44] Li E, Bestor TH, Jaenisch R (1992). Targeted mutation of the DNA methyltransferase gene results in embryonic lethality. Cell.

[CR45] Li E, Beard C, Jaenisch R (1993). Role for DNA methylation in genomic imprinting. Nature.

[CR46] Li G, Margueron R, Ku M, Chambon P, Bernstein BE, Reinberg D (2010). Jarid2 and PRC2, partners in regulating gene expression. Genes Dev.

[CR47] Lienert F, Wirbelauer C, Som I, Dean A, Mohn F, Schubeler D (2011). Identification of genetic elements that autonomously determine DNA methylation states. Nat Genet.

[CR48] Lohmann F, Loureiro J, Su H, Fang Q, Lei H, Lewis T, Yang Y, Labow M, Li E, Chen T, Kadam S (2010). KMT1E mediated H3K9 methylation is required for the maintenance of embryonic stem cells by repressing trophectoderm differentiation. Stem Cells (Dayton, Ohio).

[CR49] Majewski IJ, Ritchie ME, Phipson B, Corbin J, Pakusch M, Ebert A, Busslinger M, Koseki H, Hu Y, Smyth GK, Alexander WS, Hilton DJ, Blewitt ME (2010). Opposing roles of polycomb repressive complexes in hematopoietic stem and progenitor cells. Blood.

[CR50] Mak W, Baxter J, Silva J, Newall AE, Otte AP, Brockdorff N (2002). Mitotically stable association of polycomb group proteins eed and enx1 with the inactive x chromosome in trophoblast stem cells. Curr Biol.

[CR51] Marahrens Y, Panning B, Dausman J, Strauss W, Jaenisch R (1997). Xist-deficient mice are defective in dosage compensation but not spermatogenesis. Genes Dev.

[CR52] Meissner A, Mikkelsen TS, Gu H, Wernig M, Hanna J, Sivachenko A, Zhang X, Bernstein BE, Nusbaum C, Jaffe DB, Gnirke A, Jaenisch R, Lander ES (2008). Genome-scale DNA methylation maps of pluripotent and differentiated cells. Nature.

[CR53] Mikkelsen TS, Ku M, Jaffe DB, Issac B, Lieberman E, Giannoukos G, Alvarez P, Brockman W, Kim TK, Koche RP, Lee W, Mendenhall E, O'Donovan A, Presser A, Russ C, Xie X, Meissner A, Wernig M, Jaenisch R, Nusbaum C, Lander ES, Bernstein BE (2007). Genome-wide maps of chromatin state in pluripotent and lineage-committed cells. Nature.

[CR54] Mohn F, Weber M, Rebhan M, Roloff TC, Richter J, Stadler MB, Bibel M, Schubeler D (2008). Lineage-specific polycomb targets and de novo DNA methylation define restriction and potential of neuronal progenitors. Mol Cell.

[CR55] Nabel CS, Kohli RM (2011). Molecular biology. Demystifying DNA demethylation. Science (New York, NY).

[CR56] Nagy A, Gocza E, Diaz EM, Prideaux VR, Ivanyi E, Markkula M, Rossant J (1990). Embryonic stem cells alone are able to support fetal development in the mouse. Development.

[CR57] Ng RK, Dean W, Dawson C, Lucifero D, Madeja Z, Reik W, Hemberger M (2008). Epigenetic restriction of embryonic cell lineage fate by methylation of Elf5. Nat Cell Biol.

[CR58] Nichols J, Silva J, Roode M, Smith A (2009). Suppression of Erk signalling promotes ground state pluripotency in the mouse embryo. Development.

[CR59] Niwa H, Toyooka Y, Shimosato D, Strumpf D, Takahashi K, Yagi R, Rossant J (2005). Interaction between Oct3/4 and Cdx2 determines trophectoderm differentiation. Cell.

[CR60] O'Carroll D, Erhardt S, Pagani M, Barton SC, Surani MA, Jenuwein T (2001). The polycomb-group gene Ezh2 is required for early mouse development. Mol Cell Biol.

[CR61] Okano M, Bell DW, Haber DA, Li E (1999). DNA methyltransferases Dnmt3a and Dnmt3b are essential for de novo methylation and mammalian development. Cell.

[CR62] Orkin SH, Hochedlinger K (2011). Chromatin connections to pluripotency and cellular reprogramming. Cell.

[CR63] Pasini D, Cloos PA, Walfridsson J, Olsson L, Bukowski JP, Johansen JV, Bak M, Tommerup N, Rappsilber J, Helin K (2010). JARID2 regulates binding of the Polycomb repressive complex 2 to target genes in ES cells. Nature.

[CR64] Pastor WA, Pape UJ, Huang Y, Henderson HR, Lister R, Ko M, McLoughlin EM, Brudno Y, Mahapatra S, Kapranov P, Tahiliani M, Daley GQ, Liu XS, Ecker JR, Milos PM, Agarwal S, Rao A (2011). Genome-wide mapping of 5-hydroxymethylcytosine in embryonic stem cells. Nature.

[CR65] Pawlak M, Jaenisch R (2011). De novo DNA methylation by Dnmt3a and Dnmt3b is dispensable for nuclear reprogramming of somatic cells to a pluripotent state. Genes Dev.

[CR66] Peng JC, Valouev A, Swigut T, Zhang J, Zhao Y, Sidow A, Wysocka J (2009). Jarid2/Jumonji coordinates control of PRC2 enzymatic activity and target gene occupancy in pluripotent cells. Cell.

[CR67] Plath K, Fang J, Mlynarczyk-Evans SK, Cao R, Worringer KA, Wang H, de la Cruz CC, Otte AP, Panning B, Zhang Y (2003). Role of histone H3 lysine 27 methylation in X inactivation. Science (New York, NY).

[CR68] Poueymirou WT, Auerbach W, Frendewey D, Hickey JF, Escaravage JM, Esau L, Dore AT, Stevens S, Adams NC, Dominguez MG, Gale NW, Yancopoulos GD, DeChiara TM, Valenzuela DM (2007). F0 generation mice fully derived from gene-targeted embryonic stem cells allowing immediate phenotypic analyses. Nat Biotechnol.

[CR69] Pullirsch D, Hartel R, Kishimoto H, Leeb M, Steiner G, Wutz A (2010). The Trithorax group protein Ash2l and Saf-A are recruited to the inactive X chromosome at the onset of stable X inactivation. Development.

[CR70] Ralston A, Cox BJ, Nishioka N, Sasaki H, Chea E, Rugg-Gunn P, Guo G, Robson P, Draper JS, Rossant J (2010). Gata3 regulates trophoblast development downstream of Tead4 and in parallel to Cdx2. Development.

[CR71] Rinn JL, Kertesz M, Wang JK, Squazzo SL, Xu X, Brugmann SA, Goodnough LH, Helms JA, Farnham PJ, Segal E, Chang HY (2007). Functional demarcation of active and silent chromatin domains in human HOX loci by noncoding RNAs. Cell.

[CR72] Rossant J (2007). Stem cells and lineage development in the mammalian blastocyst. Reprod Fertil Dev.

[CR73] Sado T, Fenner MH, Tan SS, Tam P, Shioda T, Li E (2000). X inactivation in the mouse embryo deficient for Dnmt1: distinct effect of hypomethylation on imprinted and random X inactivation. Dev Biol.

[CR74] Sakaue M, Ohta H, Kumaki Y, Oda M, Sakaide Y, Matsuoka C, Yamagiwa A, Niwa H, Wakayama T, Okano M (2010). DNA methylation is dispensable for the growth and survival of the extraembryonic lineages. Curr Biol.

[CR75] Sasaki H (2010). Mechanisms of trophectoderm fate specification in preimplantation mouse development. Dev Growth Differ.

[CR76] Scheuermann JC, de Ayala Alonso AG, Oktaba K, Ly-Hartig N, McGinty RK, Fraterman S, Wilm M, Muir TW, Muller J (2010). Histone H2A deubiquitinase activity of the Polycomb repressive complex PR-DUB. Nature.

[CR77] Schoeftner S, Sengupta AK, Kubicek S, Mechtler K, Spahn L, Koseki H, Jenuwein T, Wutz A (2006). Recruitment of PRC1 function at the initiation of X inactivation independent of PRC2 and silencing. EMBO J.

[CR78] Schuettengruber B, Chourrout D, Vervoort M, Leblanc B, Cavalli G (2007). Genome regulation by polycomb and trithorax proteins. Cell.

[CR79] Seenundun S, Rampalli S, Liu QC, Aziz A, Palii C, Hong S, Blais A, Brand M, Ge K, Dilworth FJ (2010). UTX mediates demethylation of H3K27me3 at muscle-specific genes during myogenesis. EMBO J.

[CR80] Sharif J, Muto M, Takebayashi S, Suetake I, Iwamatsu A, Endo TA, Shinga J, Mizutani-Koseki Y, Toyoda T, Okamura K, Tajima S, Mitsuya K, Okano M, Koseki H (2007). The SRA protein Np95 mediates epigenetic inheritance by recruiting Dnmt1 to methylated DNA. Nature.

[CR81] Shaver S, Casas-Mollano JA, Cerny RL, Cerutti H (2010). Origin of the polycomb repressive complex 2 and gene silencing by an E(z) homolog in the unicellular alga Chlamydomonas. Epigenetics.

[CR82] Shen X, Kim W, Fujiwara Y, Simon MD, Liu Y, Mysliwiec MR, Yuan GC, Lee Y, Orkin SH (2009). Jumonji modulates polycomb activity and self-renewal versus differentiation of stem cells. Cell.

[CR83] Silva J, Nichols J, Theunissen TW, Guo G, van Oosten AL, Barrandon O, Wray J, Yamanaka S, Chambers I, Smith A (2009). Nanog is the gateway to the pluripotent ground state. Cell.

[CR84] Stock JK, Giadrossi S, Casanova M, Brookes E, Vidal M, Koseki H, Brockdorff N, Fisher AG, Pombo A (2007). Ring1-mediated ubiquitination of H2A restrains poised RNA polymerase II at bivalent genes in mouse ES cells. Nat Cell Biol.

[CR85] Tachibana M, Sugimoto K, Nozaki M, Ueda J, Ohta T, Ohki M, Fukuda M, Takeda N, Niida H, Kato H, Shinkai Y (2002). G9a histone methyltransferase plays a dominant role in euchromatic histone H3 lysine 9 methylation and is essential for early embryogenesis. Genes Dev.

[CR86] Tahiliani M, Koh KP, Shen Y, Pastor WA, Bandukwala H, Brudno Y, Agarwal S, Iyer LM, Liu DR, Aravind L, Rao A (2009). Conversion of 5-methylcytosine to 5-hydroxymethylcytosine in mammalian DNA by MLL partner TET1. Science (New York, NY).

[CR87] Tan M, Luo H, Lee S, Jin F, Yang JS, Montellier E, Buchou T, Cheng Z, Rousseaux S, Rajagopal N, Lu Z, Ye Z, Zhu Q, Wysocka J, Ye Y, Khochbin S, Ren B, Zhao Y (2011). Identification of 67 histone marks and histone lysine crotonylation as a new type of histone modification. Cell.

[CR88] Tesar PJ, Chenoweth JG, Brook FA, Davies TJ, Evans EP, Mack DL, Gardner RL, McKay RD (2007). New cell lines from mouse epiblast share defining features with human embryonic stem cells. Nature.

[CR89] Tsumura A, Hayakawa T, Kumaki Y, Takebayashi S, Sakaue M, Matsuoka C, Shimotohno K, Ishikawa F, Li E, Ueda HR, Nakayama J, Okano M (2006). Maintenance of self-renewal ability of mouse embryonic stem cells in the absence of DNA methyltransferases Dnmt1, Dnmt3a and Dnmt3b. Genes Cells.

[CR90] Voncken JW, Roelen BA, Roefs M, de Vries S, Verhoeven E, Marino S, Deschamps J, van Lohuizen M (2003). Rnf2 (Ring1b) deficiency causes gastrulation arrest and cell cycle inhibition. Proc Natl Acad Sci USA.

[CR91] Wang J, Mager J, Chen Y, Schneider E, Cross JC, Nagy A, Magnuson T (2001). Imprinted X inactivation maintained by a mouse Polycomb group gene. Nat Genet.

[CR92] Williams K, Christensen J, Pedersen MT, Johansen JV, Cloos PA, Rappsilber J, Helin K (2011). TET1 and hydroxymethylcytosine in transcription and DNA methylation fidelity. Nature.

[CR93] Wu H, D'Alessio AC, Ito S, Xia K, Wang Z, Cui K, Zhao K, Eve Sun Y, Zhang Y (2011). Dual functions of Tet1 in transcriptional regulation in mouse embryonic stem cells. Nature.

[CR94] Yamanaka S (2009). Elite and stochastic models for induced pluripotent stem cell generation. Nature.

[CR95] Yeap LS, Hayashi K, Surani MA (2009). ERG-associated protein with SET domain (ESET)-Oct4 interaction regulates pluripotency and represses the trophectoderm lineage. Epigenetics Chromatin.

[CR96] Yildirim O, Li R, Hung JH, Chen PB, Dong X, Ee LS, Weng Z, Rando OJ, Fazzio TG (2011). Mbd3/NURD complex regulates expression of 5-hydroxymethylcytosine marked genes in embryonic stem cells. Cell.

[CR97] Yuan P, Han J, Guo G, Orlov YL, Huss M, Loh YH, Yaw LP, Robson P, Lim B, Ng HH (2009). Eset partners with Oct4 to restrict extraembryonic trophoblast lineage potential in embryonic stem cells. Genes Dev.

